# Global trends and emerging insights in BRAF and MEK inhibitor resistance in melanoma: a bibliometric analysis

**DOI:** 10.3389/fmolb.2025.1538743

**Published:** 2025-01-17

**Authors:** Jianhao Bai, Zhongqi Wan, Wanru Zhou, Lijun Wang, Wei Lou, Yao Zhang, Haiying Jin

**Affiliations:** ^1^ Department of Ophthalmology, Shanghai East Hospital, Tongji University School of Medicine, Shanghai, China; ^2^ Department of Ophthalmology, Shanghai Tenth People’s Hospital Affiliated to Tongji University, Tongji University School of Medicine, Shanghai, China

**Keywords:** melanoma, therapeutic resistance, BRAF mutations, MEK inhibitors, immunotherapy

## Abstract

**Objective:**

This study aims to perform a comprehensive bibliometric analysis of global research on BRAF and MEK inhibitor resistance in melanoma, identifying key research trends, influential contributors, and emerging themes from 2003 to 2024.

**Methods:**

A systematic search was conducted in the Web of Science Core Collection (WoSCC) database to retrieve publications related to BRAF and MEK inhibitor resistance from 1 January 2003, to 1 September 2024. Bibliometric analyses, including publication trends, citation networks, and keyword co-occurrence patterns, were performed using VOSviewer and CiteSpace. Collaborative networks, co-cited references, and keyword burst analyses were mapped to uncover shifts in research focus and global cooperation.

**Results:**

A total of 3,503 documents, including 2,781 research articles and 722 review papers, were analyzed, highlighting significant growth in this field. The United States, China, and Italy led in publication volume and citation impact, with Harvard University and the University of California System among the top contributing institutions. Research output showed three phases of growth, peaking in 2020. Keyword and co-citation analyses revealed a transition from early focus on BRAF mutations and MAPK pathway activation to recent emphasis on immunotherapy, combination therapies, and non-apoptotic cell death mechanisms like ferroptosis and pyroptosis. These trends reflect the evolving priorities and innovative approaches shaping the field of resistance to BRAF and MEK inhibitors in melanoma.

**Conclusion:**

Research on BRAF and MEK inhibitor resistance has evolved significantly. This analysis provides a strategic framework for future investigations, guiding the development of innovative, multi-modal approaches to improve treatment outcomes for melanoma patients.

## Introduction

Melanoma is an aggressive and highly lethal form of skin cancer, responsible for around 60%–70% of all skin cancer-related deaths globally, despite constituting less than 5% of overall skin cancer diagnoses (Lo and Fisher, 2014; Chattopadhyay et al., 2016). The incidence of melanoma has been steadily rising, with an estimated 325,000 new cases diagnosed annually across the globe (Tagami-Nagata et al., 2015; Shain and Bastian, 2016). Among patients with metastatic melanoma, the discovery of activating mutations in the BRAF gene—particularly the BRAF V600E mutation, which occurs in approximately 40%–60% of cases—has revolutionized therapeutic strategies (Arkenau et al., 2011; Hertzman Johansson and Egyhazi Brage, 2014; Indini et al., 2019). BRAF mutations trigger the constitutive activation of the MAPK/ERK signaling pathway, promoting uncontrolled cell proliferation and tumor survival (Johnson and Dahlman, 2018). The advent of BRAF inhibitors (such as vemurafenib and dabrafenib) (Muñoz-Couselo et al., 2015)and their subsequent combination with MEK inhibitors (such as trametinib and cobimetinib) has significantly improved the prognosis for patients harboring BRAF-mutant melanoma (Bucheit and Davies, 2014; El-Nassan, 2014; Zhang, 2015). Clinical trials have demonstrated that combining BRAF and MEK inhibitors can extend progression-free survival (PFS) to 14.9 months, compared to 5.6 months for BRAF inhibitor monotherapy, while also improving overall survival (OS) (Sanchez et al., 2018). Moreover, recent advancements have demonstrated that combining BRAF/MEK inhibitors with immune checkpoint inhibitors, such as PD-1/PD-L1 inhibitors, can further enhance therapeutic efficacy by targeting complementary mechanisms, thereby overcoming resistance and improving survival outcomes in melanoma patients.

However, these therapeutic gains are frequently short-lived. 50% of patients develop resistance to BRAF and MEK inhibitors within 6–7 months of initiating treatment (Flaherty et al., 2012; Wood and Luke, 2016), underscoring the persistence of melanoma as a significant clinical challenge. Mechanistically, resistance arises through a variety of pathways (Ojha et al., 2019). Secondary mutations in NRAS, observed in 20%–30% of resistant cases, enable melanoma cells to bypass BRAF blockade by reactivating the MAPK/ERK signaling cascade, facilitating continued cell proliferation (Feng et al., 2019; Alqathama, 2020). Similarly, BRAF gene amplifications, reported in 10%–20% of resistant cases, result in the overexpression of mutant BRAF protein, driving downstream oncogenic signaling even in the presence of targeted inhibitors (Wang et al., 2021).

In addition to MAPK pathway reactivation, the activation of the PI3K/AKT/mTOR pathway frequently mediates resistance to BRAF/MEK inhibition. This pathway can act as a compensatory survival mechanism (Tehranian et al., 2022; Qi et al., 2023). PTEN loss, a tumor suppressor frequently inactivated in melanoma, exacerbates this resistance by derepressing the PI3K pathway. Moreover, the overexpression of receptor tyrosine kinases (RTKs), such as EGFR, provides melanoma cells with alternative proliferative signals that enable them to circumvent the inhibitory effects of BRAF and MEK inhibitors (Gross et al., 2015; Zhong et al., 2022). NF1 gene mutations, which occur in approximately 15% of melanomas, further enhance resistance by disinhibiting RAS, thereby sustaining MAPK pathway activation (Nissan et al., 2014). The interplay between these pathways underscores the redundancy and adaptability of melanoma cell signaling, complicating therapeutic interventions.

Ongoing research continues to elucidate these resistance mechanisms, leveraging a combination of *in vitro* studies, clinical data analysis, and molecular target screening (Johannessen et al., 2010). *In vitro* experiments using cell lines derived from resistant tumors enable researchers to manipulate specific genes and signaling pathways to investigate their roles in resistance (Delyon et al., 2023). Clinical data from patient samples are also integral, providing real-world insights into how resistance mechanisms manifest in treated individuals (Luebker and Koepsell, 2019). Advanced technologies such as next-generation sequencing (NGS) and whole-exome sequencing are frequently employed to identify secondary mutations, gene amplifications, and pathway activations associated with resistance (Wagle et al., 2014; Vergani et al., 2022). Additionally, CRISPR-based gene editing and RNA interference (RNAi) techniques help identify potential new therapeutic targets that could be exploited to overcome resistance (Corcoran et al., 2018; Redondo-Muñoz et al., 2023).

Despite significant advances in understanding the molecular underpinnings of resistance to BRAF and MEK inhibitors, there remains a gap in the quantitative analysis of research trends and collaboration patterns in this area. Bibliometric analysis offers a powerful tool for understanding the development and direction of scientific research, providing insights into publication trends, citation patterns, and global collaboration networks (Tan et al., 2023). Such analyses can identify key contributors, research hotspots, and emerging themes, guiding future research directions.

While numerous studies have explored the clinical and molecular aspects of BRAF and MEK inhibitor resistance, quantitative assessments of the global research landscape remain limited. No comprehensive bibliometric analyses currently map the evolution of research topics, highlight key research institutions, or predict emerging areas of focus in this field. This gap underscores the need for a systematic bibliometric evaluation to better understand the state of research on BRAF and MEK inhibitor resistance in melanoma, identify knowledge gaps, and inform future therapeutic strategies.

This study aims to address this critical gap by conducting a bibliometric analysis of global research on BRAF and MEK inhibitor resistance in melanoma. Through the analysis of publication trends, collaboration networks, and citation data, this research will provide a detailed overview of the field’s development, highlight influential contributors, and suggest future directions for improving treatment outcomes in melanoma patients.

## Materials and methods

### Data source and search strategy

The Web of Science Core Collection (WoSCC) database is recognized as the most reliable resource for bibliometric analysis due to its superior accuracy in categorizing and indexing publication types compared to other databases. As such, it was selected as the primary data source for this study. On 1 September 2024, a comprehensive search was conducted within WoSCC to retrieve all publications related to BRAF and MEK inhibitor resistance in melanoma. The search utilized a robust and inclusive query designed to capture a wide range of relevant studies. The specific search formula was constructed as follows: ((((((((((((((((((((((((((((((((((TS = (Vemurafenib)) OR TS = (PLX4032)) OR TS = (PLX 4032)) OR TS = (Zelboraf)) OR TS = (R05185426)) OR TS = (RG7204)) OR TS = (RG-7204)) OR TS = (RG 7204)) OR TS = (dabrafenib)) OR TS = (GSK 2118436)) OR TS = (trametinib)) OR TS = (“GSK 1120212”)) OR TS = (“GSK-1120212”)) OR TS = (“JTP 74057”)) OR TS = (“JTP-74057”)) OR TS = (JTP74057)) OR TS = (cobimetinib)) OR TS = (GDC-0973)) OR TS=(XL518)) OR TS=(“Proto-Oncogene Proteins B-raf”)) OR TS=(“B-raf, Proto-Oncogene Proteins”)) OR TS = (“Proteins B-raf, Proto-Oncogene”)) OR TS = (“Proto Oncogene Proteins B raf”)) OR TS = (“B-raf Kinase”)) OR TS = (“B raf Kinase”)) OR TS = (“Kinase, B-raf”)) OR TS = (“BRAF Kinase”)) OR TS = (“Kinase, BRAF”)) OR TS = (BRAF)) OR TS = (“MAP Kinase Kinases”)) OR TS = (“MAP3 Kinase”)) OR TS = (“MEK Kinase”)) OR TS = (“Kinase, MEK”)) OR TS = (“MEKK”)) OR TS = (MEK) AND (((((TS = (Melanoma)) OR TS = (Melanomas)) OR TS = (“Malignant Melanoma”)) OR TS = (“Malignant Melanomas”)) OR TS = (“Melanoma, Malignant”)) OR TS = (“Melanomas, Malignant”) AND (TS = (Resistance)) OR TS = (resistant).

### Inclusion and exclusion criteria

The selection of publications for this study was conducted based on the following predefined inclusion criteria: (1) full-text articles explicitly investigating resistance mechanisms to BRAF and MEK inhibitors in melanoma; (2) peer-reviewed original research articles or review papers published in the English language; and (3) publications dated between 1 January 2000, and 1 September 2024.

Exclusion criteria encompassed: (1) studies that did not directly pertain to BRAF or MEK inhibitor resistance in melanoma; and (2) non-scholarly documents, such as conference proceedings, book chapters, corrections, editorials, or news reports.

**FIGURE 1:**
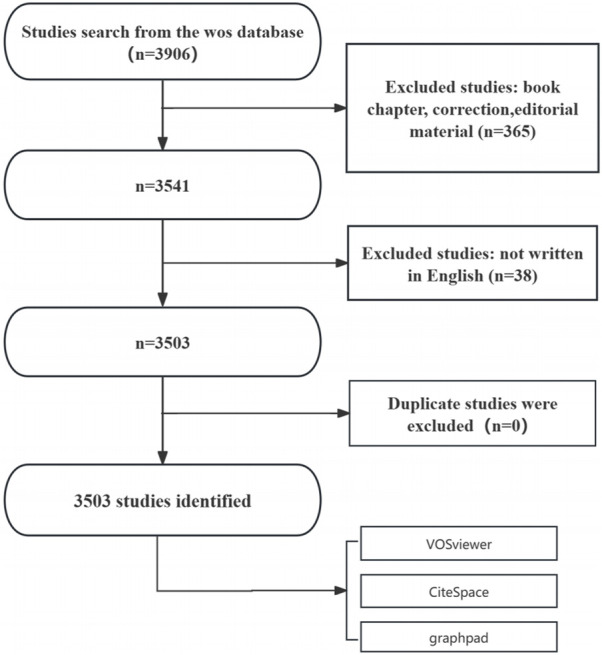
Flowchart of the publication selection process for studies on BRAF and MEK inhibitor resistance in melanoma.

Following the application of these inclusion and exclusion parameters, eligible articles were systematically exported in plain text format for further bibliometric analysis. The literature screening process is depicted in Figure 1. The initial search yielded 3,906 studies. After excluding 365 non-research materials, 3,541 studies remained. Eleven non-English articles were further excluded, resulting in 3,503 eligible studies. No duplicate records were identified.

### Data categorization and preprocessing

Data organization and preprocessing were conducted using Microsoft Excel 2021 to systematically classify and filter the retrieved publications. This process involved organizing the dataset by key bibliometric parameters, including publication year, authorship, country of origin, institutional affiliation, journal name, and citation metrics. The categorized dataset served as a structured foundation for identifying patterns and trends within the field.

Subsequent analyses were performed using the “bibliometrix” package in the R programming environment, which facilitated the generation of graphical and statistical representations of the data. These visualizations encompassed publication trends, co-authorship networks, citation analyses, and impact metrics, such as the Hirsch index (h-index).

### Data analysis and visualization

For bibliometric mapping and network analysis, we utilized VOSviewer (version 1.6.18). This software facilitated the construction of bibliometric maps to visualize co-authorship, co-citation, and keyword co-occurrence networks. The maps highlighted clusters of research activity, with node sizes representing the frequency of publications or co-occurrences. Distinct color codings were employed to differentiate thematic clusters, enabling the identification of research communities and key areas of focus. Additionally, total link strength—a metric representing the strength of connectivity between nodes—was calculated, offering insights into the collaborative structure within the field.

In parallel, we used CiteSpace (version 6.2.4R) to generate visual knowledge maps and analyze citation bursts, which signify periods of intensified research activity. CiteSpace’s co-occurrence and burst-detection capabilities allowed for the identification of pivotal shifts in research focus over time. These bursts were mapped onto a chronological timeline, providing a dynamic overview of the evolving research landscape surrounding resistance to BRAF and MEK inhibitors in melanoma. Together, these tools offered a comprehensive approach to analyzing and visualizing the intellectual structure and trends within the field.

### Ethical considerations

This study is based solely on publicly available data from the WoSCC database. Since no human or animal subjects were involved, ethical approval was not required. All analyses were conducted in accordance with established bibliometric research practices.

## Results

### Summary

The search results revealed that a total of 3,503 documents related to BRAF and MEK inhibitor resistance in melanoma were indexed. This collection comprised 2,781 research articles (79.37%) and 722 review papers (20.63%), indicating a substantial body of original research supported by comprehensive reviews. These publications were contributed by researchers from 86 countries and regions, 3,173 institutions, and involved 20,081 individual authors.

**FIGURE 2:**
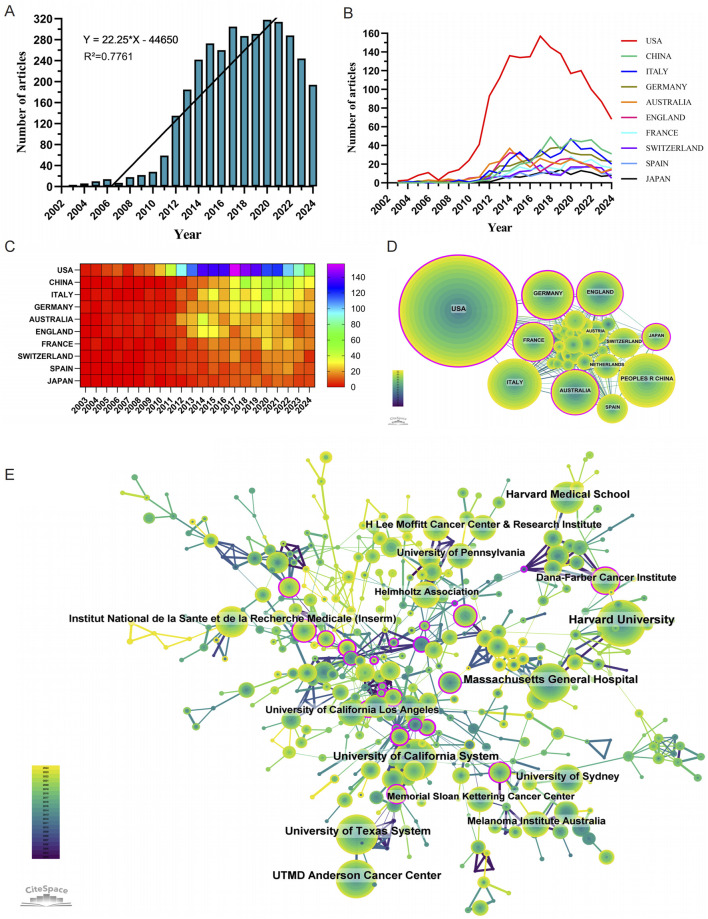
Comprehensive analysis of global research trends in BRAF and MEK inhibitor resistance in melanoma: Publication Dynamics, Collaborations, and Major Contributors (2003–2024). **(A)** Annual number of publications on BRAF and MEK inhibitor resistance in melanoma from 1 January 2003 to 31 December 2024. **(B, C)** Line chart **(B)** and heatmap **(C)** illustrating the annual publication volume of the top 10 countries over the past 2 decades. **(D)** Global collaboration network in BRAF and MEK inhibitor resistance research in melanoma. **(E)** Institutional contributions to research on BRAF and MEK inhibitor resistance in melanoma.

The annual number of publications has shown a consistent upward trend since 2003 (Figure 2A). During the first phase (2003–2007), fewer than 15 papers were published annually, representing a relatively slow growth rate of approximately 3% per year. In the second phase (2008–2011), annual publications increased to an average of 50 papers per year, reflecting a substantial growth rate of approximately 233% compared to the first phase. The third phase (2012 onward) saw an exponential rise, with publications increasing from 135 in 2012 to a peak of 406 in 2020—an annual growth rate of approximately 20% during this period. These patterns underscore the progressive development and global expansion of research on resistance to BRAF and MEK inhibitors in melanoma.

### Countries and institutions

Research on BRAF and MEK inhibitor resistance in melanoma has been conducted across 86 countries and regions. Figures 2B, C illustrate the annual publication trends over the past decade for the top 10 countries in this field. The top five countries by publication volume are the United States, China, Italy, Germany, and Australia. Among these, the United States dominates, contributing 47.30% of all publications, far surpassing other nations.

In terms of citation impact, the United States leads with a total of 144,860 citations (Table 1), reflecting a high level of academic influence. Its citation-to-publication ratio of 87.42 ranks among the highest globally, underscoring the consistently high quality of its research output. China, which ranks second in publication volume (393 papers), has accrued 10,884 citations, placing it ninth in total citations. However, its citation-to-publication ratio of 27.69 is comparatively lower, indicating room for improvement in the impact of its research output. The collaboration network (Figure 2D) shows that the United States maintains strong collaborative ties with Germany, France, and Italy, while China collaborates most frequently with Spain, Australia, and Japan. Notably, United States’s network centrality of 0.37 indicates its emerging leadership role in the field, driven by both publication volume and collaboration strength.

**TABLE 1:** The top 10 most productive countries in BRAF and MEK inhibitor resistance research in melanoma.

Rank	Country/region	Article counts	Centrality	Percentage (%)	Citation	Citation per publication
1	United States	1,657	0.37	47.30%	144,860	87.42
2	China	393	0.09	11.22%	10,884	27.69
3	Italy	375	0.06	10.71%	29,420	78.45
4	Germany	345	0.26	9.85%	31,453	91.17
5	Australia	302	0.13	8.62%	42,778	141.65
6	England	283	0.15	8.08%	30,101	106.36
7	France	218	0.12	6.22%	26,584	121.94
8	Switzerland	157	0.04	4.48%	23,768	151.39
9	Spain	142	0.07	4.05%	15,710	110.63
10	Japan	112	0.15	3.20%	6,208	55.43

A total of 3,173 institutions have systematically contributed to the research on BRAF and MEK inhibitor resistance. Among the top 10 institutions by publication volume, seven are from the United States, two are from Australia, and one is from France (Table 2; Figure 2E). Harvard University ranks first with 327 publications, 55,154 citations, and an average of 168.67 citations per paper. The University of California System ranks second with 242 publications, 49,179 citations, and an impressive average of 203.22 citations per paper. The University of Texas System follows with 240 publications, 24,735 citations, and an average of 103.06 citations per paper, while Massachusetts General Hospital ranks fourth with 222 publications, 41,884 citations, and an average of 188.67 citations per paper.

**TABLE 2:** The top 10 institutions publishing literature in BRAF and MEK inhibitor resistance research in melanoma.

Rank	Institution	Country	Number of studies	Total citations	Average citation
1	Harvard University	United States	327	55,154	168.67
2	University of California System	United States	242	49,179	203.22
3	University of Texas System	United States	240	24,735	103.06
4	Massachusetts General Hospital	United States	222	41,884	188.67
5	UTMD Anderson Cancer Center	United States	213	23,909	112.25
6	Harvard Medical School	United States	181	26,668	147.34
7	Institut National de la Sante et de la Recherche Medicale (Inserm)	France	151	10,569	69.99
8	University of Sydney	Australia	138	21,370	154.86
9	University of California Los Angeles	United States	118	37,304	316.14
10	Melanoma Institute Australia	Australia	114	16,241	142.46

Further analysis reveals that institutions, both domestically and internationally, predominantly collaborate with other institutions within their own countries. This trend highlights strong intra-national academic networks but also underscores the need for enhanced international collaboration. We advocate for stronger partnerships between institutions across borders to break down academic barriers and foster the global exchange of knowledge, which is critical for advancing research on BRAF and MEK inhibitor resistance in melanoma.

## Journal

**FIGURE 3:**
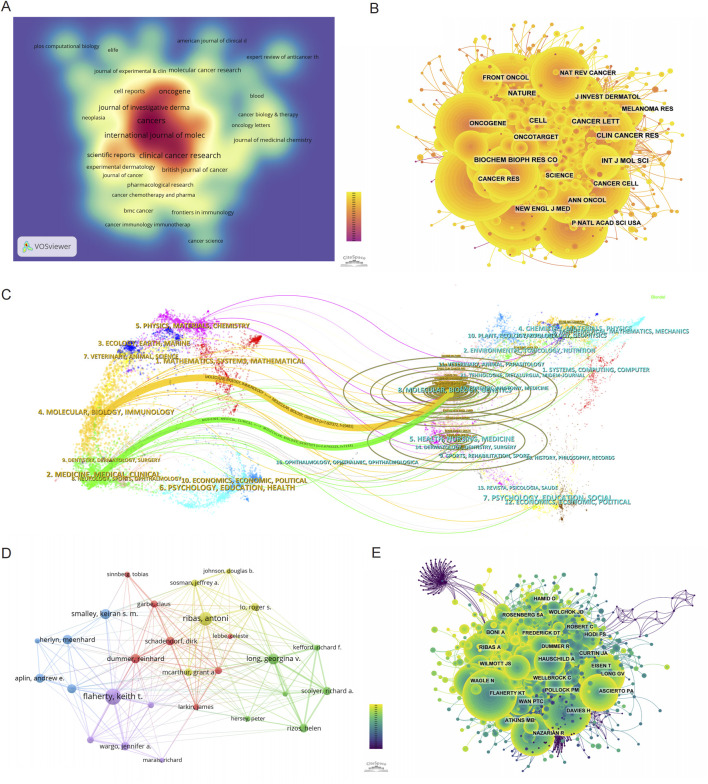
Mapping the research landscape of BRAF and MEK inhibitor resistance in melanoma: Publication Density, Journal Networks, and Author Collaborations. **(A)** Density map of journal publications in the field of BRAF and MEK inhibitor resistance in melanoma. **(B)** Network map showing co-cited journals in this research area. **(C)** Dual-map overlay of journals related to BRAF and MEK inhibitor resistance, with colored tracks representing citation pathways, showing citing journals on the left and cited journals on the right. **(D)** Author collaboration network, highlighting the most prolific authors and their research collaborations in BRAF and MEK inhibitor resistance in melanoma. **(E)** Author co-citation network map illustrating the most frequently co-cited authors in this field.

Tables 3, 4 summarize the top 10 journals by publication volume and citation impact in the field of BRAF and MEK inhibitor resistance in melanoma. Among these, Cancers is the most prolific journal, contributing 145 publications (4.14%), followed by Clinical Cancer Research with 121 publications (3.45%), Oncotarget with 112 publications (3.20%), and the International Journal of Molecular Sciences with 101 publications (2.88%) (Figure 3A). Notably, among the top 10 most productive journals, Cancer Discovery has the highest impact factor (IF) of 29.7, signifying its considerable influence within the field. Furthermore, 90% of these journals are categorized within the Q1 quartile, reflecting their strong standing in the academic community.

**TABLE 3:** The top 10 productive journals in BRAF and MEK inhibitor resistance research in melanoma.

Rank	Journal	Article counts	Percentage (3,503)	If	Quartile in category
1	Cancers	145	4.14%	4.5	Q1
2	Clinical cancer research	121	3.45%	10.0	Q1
3	Oncotarget	112	3.20%	—	—
4	International journal of molecular sciences	101	2.88%	4.9	Q1
5	Cancer research	96	2.74%	12.5	Q1
6	Molecular cancer therapeutics	79	2.26%	5.3	Q1
7	Oncogene	78	2.23%	6.9	Q1
8	Pigment cell and melanoma research	63	1.80%	3.9	Q1
9	Cancer discovery	60	1.71%	29.7	Q1
10	Plos one	54	1.54%	2.9	Q1

**TABLE 4:** The top 10 co-cited journals in BRAF and MEK inhibitor resistance research in melanoma.

Rank	Cited journal	Co-citation	IF (2023)	Quartile in category
1	Nature	2,918	50.5	Q1
2	Cancer res	2,727	12.5	Q1
3	New engl j med	2,641	96.2	Q1
4	Clin cancer res	2,531	10.0	Q1
5	Cell	2,177	45.5	Q1
6	J clin oncol	2099	42.1	Q1
7	P natl acad sci United States	2077	9.4	Q1
8	Cancer cell	2034	48.8	Q1
9	Oncogene	1929	6.9	Q1
10	Cancer discov	1869	29.7	Q1

The broader influence of journals in this field was assessed through co-citation analysis, which evaluates the frequency with which journals are cited together. As shown in Figure 3B and Table 4, Nature is the most frequently co-cited journal, with 2,918 co-citations, followed by Cancer Research (2,727 co-citations) and the New England Journal of Medicine (NEJM) (2,641 co-citations). Among the top 10 co-cited journals, NEJM has the highest impact factor (IF) of 96.2, highlighting its exceptional influence on the scientific discourse in this field. Importantly, all of the top 10 co-cited journals are categorized in the Q1 quartile, underscoring their central role in shaping the research landscape of BRAF and MEK inhibitor resistance in melanoma.

The thematic distribution and citation dynamics of academic publications are visualized using a dual-map overlay (Figure 3C). This analysis reveals two primary citation pathways: research published in journals within the Molecular/Biology/Genetics domain is predominantly cited by journals within the Molecular/Biology/Immunology and Medicine/Medical/Clinical domains. The colored trajectories in the dual-map overlay illustrate these citation relationships, indicating that findings in BRAF and MEK inhibitor resistance contribute significantly to broader advancements in molecular biology, immunology, and clinical medicine. These cross-disciplinary citation patterns emphasize the wide-ranging impact of research in this field, extending its influence beyond melanoma studies to inform related areas of biological and medical sciences.

### Authors and co-citing authors

Among all authors contributing to research on BRAF and MEK inhibitor resistance in melanoma, the top 10 authors by publication volume are listed in Table 5. Collectively, these authors have published 510 papers, accounting for 14.56% of the total publications in this field. Flaherty, Keith T. leads the list with 80 publications, followed by Ribas, Antoni (67 publications) and Long, Georgina V. (54 publications). The collaborative network among these authors, visualized using CiteSpace (Figure 3D), reveals tightly knit clusters, with Flaherty, Keith T. occupying a central position, indicating his strong collaborative ties with other prominent researchers in the field.

**TABLE 5:** Top 10 most prolific and co-cited authors in BRAF and MEK inhibitor resistance research in melanoma.

Rank	Author	Count	Rank	Co-cited author	Citation
1	Flaherty, keith t	80	1	Flaherty kt	1,376
2	Ribas, antoni	67	2	Chapman pb	1,270
3	Long, georgina v	54	3	Davies h	1,184
4	Smalley, keiran s. M	50	4	Long gv	1,068
5	Dummer, reinhard	45	5	Nazarian r	917
6	Herlyn, meenhard	45	6	Robert c	875
7	Aplin, andrew e	44	7	Poulikakos pi	844
8	Davies, michael a	43	8	Shi hb	757
9	Rizos, helen	41	9	Hauschild a	737
10	Schadendorf, dirk	41	10	Villanueva j	716

In addition to publication output, co-citation analysis sheds light on the broader impact of these authors' work. Figure 3E and Table 6 highlight the top 10 most frequently co-cited authors. A total of 194 authors have been co-cited more than 50 times, demonstrating their substantial influence within the research community. The largest nodes in the co-citation network correspond to Flaherty, Keith T. (1,376 co-citations), Chapman, PB (1,270 co-citations), and Davies, H (1,184 co-citations). These authors' work is foundational to the understanding of BRAF and MEK inhibitor resistance, as evidenced by their central positions in the co-citation network.

**TABLE 6:** Top 10 co-cited references with the highest centrality in BRAF and MEK inhibitor resistance research in melanoma.

Rank	Title	Journal	author(s)	Total citations
1	Improved survival with vemurafenib in melanoma with BRAF V600E Mutation	New england journal of medicine	Chapman PB	628
2	Improved survival with MEK Inhibition in BRAF-mutated melanoma	New england journal of medicine	Flaherty KT	434
3	Melanomas acquire resistance to B-RAF(V600E) inhibition by RTK or N-RAS upregulation	Nature	Nazarian R	429
4	Inhibition of mutated, activated BRAF in metastatic melanoma	New england journal of medicine	Flaherty KT	414
5	Dabrafenib in BRAF-mutated metastatic melanoma: a multicentre, open-label, phase 3 randomised controlled trial	Lancet	Hauschild A	389
6	Survival in BRAF V600-mutant advanced melanoma treated with vemurafenib	New england journal of medicine	Sosman JA	376
7	COT drives resistance to RAF inhibition through MAP kinase pathway reactivation	Nature	Johannessen CM	315
8	RAF inhibitor resistance is mediated by dimerization of aberrantly spliced BRAF (V600E)	Nature	Poulikakos PI	310
9	Acquired resistance to BRAF inhibitors mediated by a RAF kinase switch in melanoma can be overcome by cotargeting MEK and IGF-1R/PI3K	Cancer cell	Villanueva J	309
10	Improved Overall Survival in Melanoma with Combined Dabrafenib and Trametinib	New england journal of medicine	Robert C	305

A deeper analysis of publication volume and citation impact further underscores the leadership of Flaherty, Keith T., who ranks first in both metrics. His prolific output and high citation counts affirm his pivotal role as a leading figure in the field, making significant contributions to advancing research and shaping the academic discourse on BRAF and MEK inhibitor resistance in melanoma.

### Co-citation of references

**FIGURE 4:**
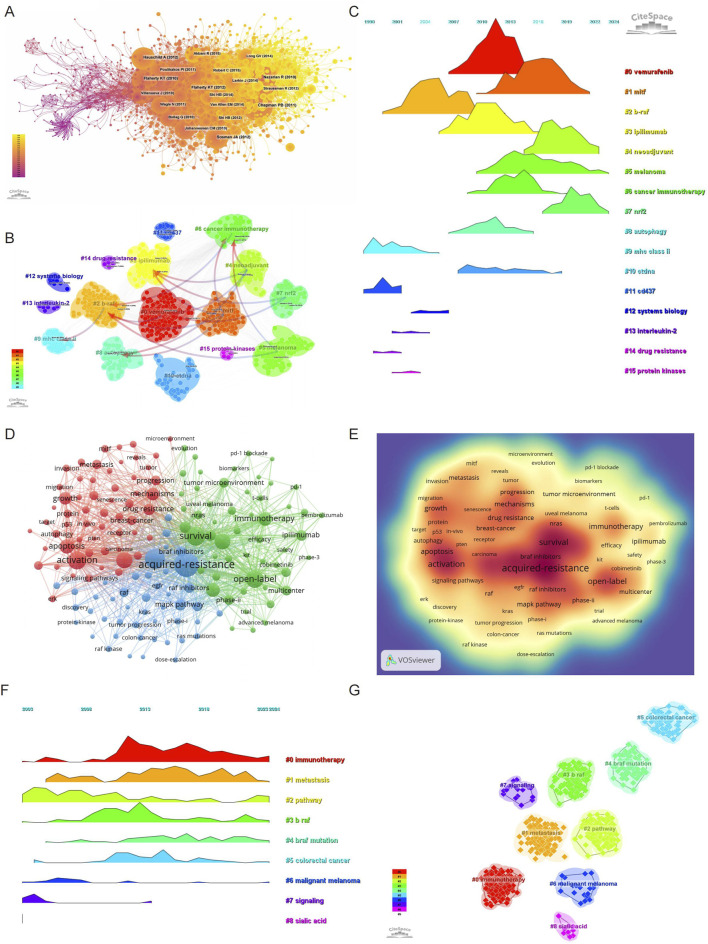
Evolution and impact of research on BRAF and MEK inhibitor resistance in melanoma: Co-cited Literature, Keywords, and Trends (2003–2024). **(A)** Co-cited literature network map showing foundational studies in BRAF and MEK inhibitor resistance. **(B)** Cluster analysis of co-cited references in the field. **(C)** Temporal distribution of co-cited references illustrating the evolution of research topics over time, highlighting shifts in research focus and emerging trends. **(D)** Network diagram highlighting high-frequency keywords in BRAF and MEK inhibitor resistance research. **(E)** Keyword density map showing the concentration of key topics in BRAF and MEK inhibitor resistance research. **(F)** Temporal heatmap displaying the progression of key research areas from 2003 to 2024, with color-coded clusters representing distinct research themes. **(G)** Clustering analysis of research hotspots in BRAF and MEK inhibitor resistance in melanoma.

Using 1-year time slices from 2003 to 2024, a co-citation reference network was constructed, comprising 1,759 nodes and 9,758 links (Figure 4A). The top 10 most frequently co-cited references are listed in Table 6. The most co-cited reference is the article titled “Improved Survival with Vemurafenib in Melanoma with BRAF V600E Mutation,” published in the New England Journal of Medicine by Chapman et al. (2011). This study demonstrated the efficacy of vemurafenib (PLX4032), a selective BRAF kinase inhibitor, in treating metastatic melanoma with the BRAF V600E mutation. Compared to dacarbazine, vemurafenib significantly improved overall survival (OS) and progression-free survival (PFS), achieving a 6-month OS rate of 84% *versus* 64%, and a median PFS of 5.3 months *versus* 1.6 months. The study also identified a confirmed objective response rate of 48% for vemurafenib compared to 5% for dacarbazine, emphasizing its efficacy in tumor control. However, the study acknowledged the emergence of therapeutic resistance, primarily through reactivation of the MAPK/ERK pathway, which limits long-term benefits. These findings have profoundly influenced research on BRAF inhibitor resistance, serving as a pivotal reference for understanding therapeutic challenges and guiding the development of combination strategies to overcome resistance mechanisms.

The second most co-cited reference is the article “Improved Survival with MEK Inhibition in BRAF-Mutated Melanoma,” authored by Flaherty et al., also published in the New England Journal of Medicine (Flaherty et al., 2012). This studyprovides pivotal evidence for the clinical efficacy of trametinib, a selective MEK1/2 inhibitor, in treating metastatic melanoma with BRAF V600E or V600K mutations. This phase 3 open-label trial demonstrated that trametinib significantly improved progression-free survival (PFS) (median PFS of 4.8 months vs 1.5 months; hazard ratio for progression, 0.45; P < 0.001) and overall survival (OS) at 6 months (81% vs 67%; hazard ratio for death, 0.54; P = 0.01) compared to chemotherapy. Trametinib’s ability to selectively inhibit MEK activity downstream of BRAF mutations underscores its role in reducing oncogenic MAPK pathway signaling. The study also highlighted trametinib’s acceptable safety profile, with manageable adverse events such as rash, diarrhea, and peripheral edema, and no reported cases of secondary squamous-cell carcinomas, which are frequently associated with BRAF inhibitors. The study highlighted the emerging role of MEK inhibitors in addressing resistance to BRAF-targeted therapies, forming the basis for combination approaches in melanoma treatment. These findings remain central to understanding and overcoming therapeutic resistance in melanoma.

A clustering and temporal analysis of co-cited references (Figures 4B, C) further revealed distinct research hotspots over time. Early research focused on topics such as mhc class ii (cluster 9), cd437 (cluster 11), interleukin-2 (cluster 13), drug resistance (cluster 14), and protein kinase (cluster 15), reflecting foundational studies on melanoma immunology and resistance pathways. Mid-phase research hotspots included vemurafenib (cluster 0), ipilimumab (cluster 3), and autophagy (cluster 8), highlighting the expanding focus on targeted therapies and immune checkpoint inhibitors.

Recent research trends have shifted toward advanced and emerging topics such as mitf (cluster 1), neoadjuvant (cluster 4), melanoma (cluster 5), cancer immunotherapy (cluster 6), nrf2 (cluster 7), and ctDNA (cluster 10). These clusters represent cutting-edge investigations into novel biomarkers, genetic mechanisms, and therapeutic strategies aimed at addressing the limitations of existing treatments.

The timeline analysis in Figure 4C visually illustrates the progression and evolution of these research clusters. This transition from early explorations of fundamental molecular mechanisms to advanced studies on therapeutic resistance and novel intervention strategies underscores the dynamic and innovative nature of research in this field. These findings provide a comprehensive overview of the research trajectory and highlight the current and emerging trends that shape the field of BRAF and MEK inhibitor resistance in melanoma.

### Keyword analysis

Keyword analysis provides a comprehensive overview of the research landscape and emerging trends in BRAF and MEK inhibitor resistance in melanoma. Using VOSviewer, we analyzed the co-occurrence of keywords, identifying acquired-resistance as the most frequently occurring term (748 occurrences), followed by mutations (513), survival (461), activation (424), and open-label (411) (Table 7; Figures 4D, E). After removing irrelevant terms, we constructed a network of 156 keywords, each appearing at least 31 times, resulting in three distinct clusters that highlight different research focuses.

**TABLE 7:** Top 10 most frequent and central keywords in BRAF and MEK inhibitor resistance research in melanoma.

Rank	Keyword	Counts	Rank	Keyword	Counts
1	Acquired-resistance	748	11	Mechanisms	225
2	Mutations	513	12	Growth	213
3	Survival	461	13	Drug resistance	178
4	Activation	424	14	Mapk pathway	171
5	Open-label	411	15	Cutaneous melanoma	170
6	Pathway	349	16	Combination	151
7	Targeted therapy	331	17	Ipilimumab	151
8	Apoptosis	266	18	Braf inhibitors	138
9	Improved survival	261	19	Raf inhibitors	138
10	Immunotherapy	249	20	Progression	135

Cluster 1 (Red) contains 58 keywords and focuses on the molecular mechanisms underlying resistance. Representative terms include activation, mechanism, drug resistance, autophagy, metabolism, oxidative stress, microenvironment, therapeutic factor, and protein. This cluster reflects research aimed at understanding the biological and molecular pathways contributing to resistance and identifying therapeutic targets. Cluster 2 (Green) comprises 53 keywords emphasizing clinical application and therapeutic approaches. Key terms include survival, open-label, safety, immunotherapy, PD-1, blockade, biomarkers, chemotherapy, immune checkpoint inhibitor, and combination therapy. This cluster underscores efforts to improve survival through novel therapies and combination strategies. Cluster 3 (Blue) includes 45 keywords related to resistance mechanisms and signaling pathways. Notable terms include mutation, acquired resistance, MAPK pathway, amplification, dose escalation, RAS mutation, solid tumor, and phase I. This cluster highlights research on resistance mechanisms, particularly focusing on pathway reactivation and strategies to overcome resistance.

To visualize the temporal evolution of research hotspots, we utilized CiteSpace to generate a heatmap of keyword trends (Figures 4F, G). Over time, terms such as immunotherapy, metastasis, pathway, BRAF, BRAF mutation, colorectal cancer, and malignant melanoma have emerged as prominent research themes. These findings indicate a growing focus on understanding resistance mechanisms, exploring immunotherapeutic strategies, and expanding the application of targeted therapies to other cancers.

This keyword analysis provides critical insights into the progression of research in BRAF and MEK inhibitor resistance, highlighting key areas of focus and potential directions for future investigation. By understanding these trends, researchers can better identify gaps in the field and develop innovative strategies to address the challenges of therapeutic resistance.

### Emerging trends and new developments

**FIGURE 5:**
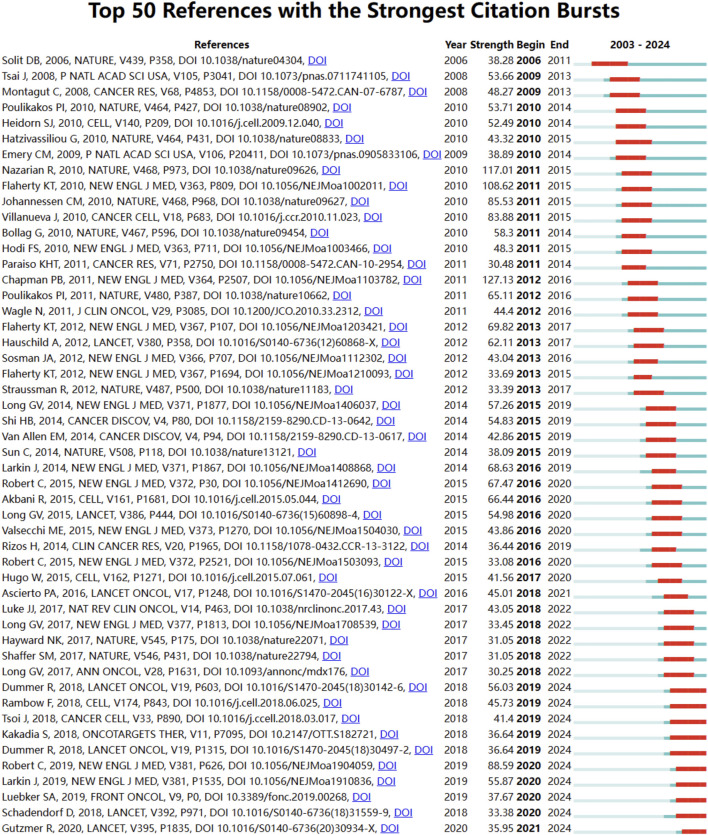
Reference burst analysis in BRAF and MEK inhibitor resistance research in melanoma from 2003 to 2024. The blue lines represent the timeline, while the red sections highlight periods of intense citation activity for key references.

Using CiteSpace, we identified the top 50 references with the strongest citation bursts in the field of BRAF and MEK inhibitor resistance in melanoma (Figure 5). The reference with the highest burst strength (45.52) is the seminal article “Improved Survival with Vemurafenib in Melanoma with BRAF V600E Mutation,” published in the New England Journal of Medicine by Paul B. Chapman et al. (Chapman et al., 2011). The identified 50 references, published between 2003 and 2024, highlight their lasting influence on the field over the past 2 decades. Notably, 10 of these references are currently at their citation peak, indicating sustained interest and relevance in the area of resistance mechanisms and therapeutic innovation for melanoma.

**FIGURE 6:**
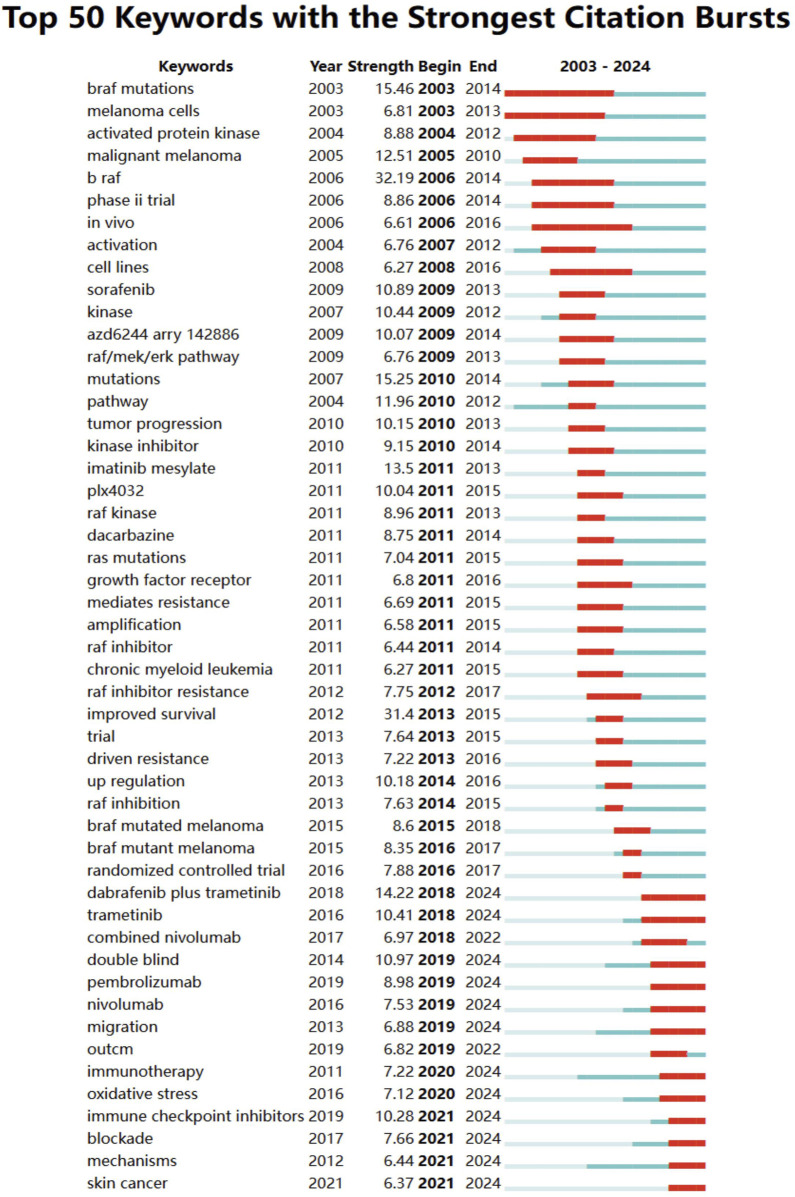
Keyword burst analysis in BRAF and MEK inhibitor resistance research in melanoma from 2003 to 2024. Keywords with the strongest citation bursts are displayed, reflecting emerging research trends and potential future directions in the field.

In addition to references, we analyzed 756 keywords with citation bursts and focused on the 50 strongest bursts (Figure 6). Keywords such as immunotherapy, BRAF mutations, pathway, malignant melanoma, RAF inhibition, and immune checkpoint inhibitors have emerged as central themes in the field.

Early research focused on BRAF mutations, malignant melanoma, and activation, emphasizing foundational studies on molecular pathways and therapeutic targets. Later trends shifted toward immunotherapy, combination therapies, checkpoint inhibitors, and oxidative stress, showcasing the field’s progression toward innovative treatment strategies. Recent bursts include nivolumab, pembrolizumab, and double-blind trials, reflecting the growing focus on advanced clinical trials and immunotherapeutic approaches. These keywords represent evolving priorities, with a clear focus on overcoming resistance mechanisms and improving outcomes through targeted therapy, combination regimens, and immune-based strategies. Together, these findings underscore the dynamic nature of the research landscape and point to critical areas of focus for future studies on BRAF and MEK inhibitor resistance in melanoma.

## Discussion

This study provides a comprehensive bibliometric analysis of global research on BRAF and MEK inhibitor resistance in melanoma, highlighting key trends, influential contributors, and emerging research priorities. The findings reveal a consistent increase in publication output over the past 2 decades, underscoring the growing academic and clinical interest in overcoming resistance to targeted therapies. The United States, China, and Italy emerge as the most prolific contributors, with institutions such as Harvard University and the University of California System leading the field in publication volume and citation impact. Collaborative networks illustrate strong international partnerships, particularly between the United States and European countries, as well as China’s increasing integration into global research efforts.

Through keyword analysis, we identified prominent themes that reflect the evolving research landscape. Early research in the field primarily centered on understanding BRAF mutations and their role in activating the MAPK/ERK signaling pathway, laying the groundwork for the development of BRAF and MEK inhibitors (Cantini et al., 2009; Mologni et al., 2018; Poulikakos et al., 2022). These foundational studies demonstrated significant therapeutic advancements, particularly with the introduction of selective inhibitors targeting BRAF V600E mutations, as evidenced by landmark clinical trials (Kopetz et al., 2019; Ascierto et al., 2023). However, as therapeutic resistance emerged as a critical challenge, the focus of research shifted toward identifying mechanisms of resistance, including pathway reactivation and alternative signaling pathway activation, such as the PI3K/AKT/mTOR pathway (Orgaz et al., 2020; Hanrahan et al., 2024).

Recent studies, as highlighted by this bibliometric analysis, indicate a growing emphasis on immunotherapy, combination therapies, and emerging non-apoptotic cell death mechanisms such as ferroptosis and pyroptosis (Du et al., 2021; Lester et al., 2023; Liu et al., 2023; Sun et al., 2023). These approaches represent an evolution in strategy, addressing the limitations of monotherapy by integrating immune checkpoint inhibitors and exploring alternative cellular vulnerabilities to enhance therapeutic efficacy. The increasing focus on keywords like immune checkpoint blockade (Amaria et al., 2018; Auslander et al., 2018), oxidative stress (Piskounova et al., 2015; Tsoi et al., 2018), and tumor microenvironment (Marzagalli et al., 2019; Han et al., 2021) reflects the field’s shift toward multidisciplinary approaches that aim to overcome resistance and improve long-term outcomes in melanoma patients (Marzagalli et al., 2019). The prominence of “immune checkpoint blockade” reflects significant breakthroughs such as the FDA approval of immune checkpoint inhibitors (ICIs) like nivolumab and pembrolizumab, which have transformed the melanoma treatment landscape by reactivating exhausted T cells and enhancing anti-tumor immunity. Combination strategies involving ICIs and BRAF/MEK inhibitors have further advanced this approach, highlighting its clinical relevance. Similarly, the increasing focus on “oxidative stress” can be attributed to discoveries linking reactive oxygen species to cancer cell survival and resistance mechanisms. These breakthroughs underscore the dynamic evolution of melanoma research, with a growing emphasis on multidisciplinary and mechanistically informed therapeutic strategies.

The resistance mechanisms underlying BRAF and MEK inhibitor therapy in melanoma are multifaceted and involve complex molecular pathways that significantly limit the durability of therapeutic responses. One of the most well-characterized mechanisms is the reactivation of the MAPK/ERK pathway, which occurs through secondary mutations in upstream regulators, such as NRAS, or through BRAF amplification (Yin et al., 2019; Randic et al., 2021). These alterations enable melanoma cells to bypass BRAF inhibition, leading to sustained signaling that promotes tumor growth and survival (Hodis et al., 2012). The significance of this mechanism is underscored by its consistent appearance in both co-cited references and high-frequency keywords such as MAPK pathway, reactivation, and mutations, reflecting its central role in therapeutic resistance research.

Another critical pathway implicated in resistance is the PI3K/AKT/mTOR signaling axis, often activated by PTEN loss or receptor tyrosine kinase (RTK) upregulation, such as EGFR or MET amplification (Tehranian et al., 2022; Geng et al., 2023; Qi et al., 2023). These alterations drive tumor survival independently of MAPK signaling, highlighting the compensatory interplaybetween these pathways (Sun et al., 2014). The integration of PI3K/AKT pathway research into therapeutic strategies is evident from the increasing focus on combination treatments targeting both the MAPK and PI3K/AKT pathways, as identified through co-occurrence analyses of keywords like combination therapy and targeted therapy.

Emerging research also highlights the role of immune evasion mechanisms in therapeutic resistance, particularly in the context of immunotherapy (Eddy and Chen, 2020; Leuzzi et al., 2024). Melanoma cells adapt to immune pressure by modulating the tumor microenvironment, including the upregulation of immune checkpoint molecules such as PD-L1 or by promoting a suppressive microenvironment through the recruitment of regulatory T cells (Tregs) and myeloid-derived suppressor cells (MDSCs) (Liu et al., 2020; Liu et al., 2023). Tregs inhibit the activation and proliferation of effector T cells, thereby reducing anti-tumor immune responses, while MDSCs suppress immunity through the production of inhibitory cytokines like IL-10 and TGF-β. Additionally, these cells promote an immunosuppressive milieu by upregulating PD-L1 expression on tumor and stromal cells, further reducing the efficacy of PD-1 inhibitors. Recent studies have indicated that Notch4 mutations may serve as predictive biomarkers for melanoma immunotherapy (Long et al., 2021; Li et al., 2022). These mutations are thought to influence immune responses by modulating the formation of lymphatic vessels in the dermis of murine models, which could impact immune cell trafficking and the efficacy of immunotherapies (Muley et al., 2022). Notch4’s regulation of lymphangiogenesis may enhance the ability of tumors to escape immune surveillance, thus affecting treatment outcomes in melanoma. These findings, frequently cited and analyzed in keywords such as immune checkpoint blockade and tumor microenvironment, underscore the necessity of integrating immune-based therapies with targeted inhibitors to overcome resistance.

These resistance mechanisms collectively illustrate the intricate interplay between oncogenic pathways and the tumor microenvironment, complicating treatment strategies. The prominence of these topics in bibliometric analyses, as reflected in keyword clusters and co-citation networks, highlights their importance as research priorities. Understanding these mechanisms has directly influenced the design of current therapeutic strategies, such as the development of triplet therapies combining BRAF and MEK inhibitors with immune checkpoint inhibitors or PI3K/AKT inhibitors (Arangalage et al., 2021; Hong et al., 2022). Such approaches aim to simultaneously target multiple vulnerabilities, providing a more robust and durable therapeutic response. As these resistance mechanisms continue to evolve, their elucidation will remain critical for advancing melanoma treatment paradigms.

The integration of ICIs, such as PD-1/PD-L1 inhibitors, with BRAF/MEK inhibitors represents a transformative approach in melanoma treatment (Gide et al., 2018; Carlino et al., 2021). This combination therapy aims to address the limitations of monotherapy by simultaneously targeting tumor-specific signaling pathways and enhancing anti-tumor immune responses. Clinical trials have demonstrated that the combination of ICIs (e.g., nivolumab or pembrolizumab) with BRAF/MEK inhibitors prolongs progression-free survival and overall survival by mitigating resistance mechanisms associated with monotherapies (Queirolo et al., 2019; Robert et al., 2019; Sabbatino et al., 2022). These results are supported by our bibliometric findings, which highlight the increasing prominence of keywords such as immune checkpoint blockade, combination therapy, and tumor microenvironment as pivotal research themes.

The rationale for combining these modalities lies in their complementary mechanisms of action. While BRAF/MEK inhibitors reduce tumor burden by directly targeting the MAPK pathway, ICIs reinvigorate exhausted T cells and counteract immune evasion mechanisms, such as PD-L1 overexpression and the recruitment of suppressive immune cells like regulatory T cells (Tregs) and myeloid-derived suppressor cells (MDSCs) (Oliveira et al., 2022). Notably, the dual targeting of the tumor microenvironment and oncogenic signaling pathways disrupts feedback loops that would otherwise enable tumor survival and resistance. Recent studies also suggest that BRAF/MEK inhibition can enhance tumor immunogenicity by increasing the presentation of tumor antigens, providing further synergy with ICIs (Razavi et al., 2021; Ghasemi et al., 2024).

Beyond immune checkpoint inhibitors, emerging therapeutic approaches such as gene therapy, cancer vaccines, and oncolytic viruses are gaining traction (Blass and Ott, 2021; North et al., 2022; Shalhout et al., 2023). For example, the use of CRISPR/Cas9-based gene editing to disrupt resistance-associated genes or modulate immune cell function represents a promising avenue (Hung et al., 2022). Similarly, personalized cancer vaccines that target neoantigens specific to BRAF-mutated melanoma are under investigation. These innovative strategies, when integrated with ICIs and targeted therapies, hold the potential to revolutionize melanoma treatment by providing highly individualized, multi-modal solutions.

Keyword burst analysis highlights a dynamic shift in research priorities over time. Early studies predominantly focused on apoptosis and MAPK pathway activation, reflecting the initial exploration of targeted therapies. However, recent trends emphasize non-apoptotic cell death mechanisms such as ferroptosis and pyroptosis, as well as the pivotal role of the tumor microenvironment (Al Mamun et al., 2021; Wang et al., 2023). Ferroptosis, a regulated form of lipid peroxidation-dependent cell death, has been implicated in overcoming resistance to targeted therapies. Similarly, pyroptosis, an inflammatory form of cell death driven by gasdermin activation, has been shown to synergize with immunotherapy by promoting the release of tumor antigens and pro-inflammatory cytokines (Khan et al., 2022). The growing focus on these topics reflects a paradigm shift in melanoma research, moving beyond single-pathway inhibition toward more comprehensive strategies that address the interplay between oncogenic signaling, immune regulation, and cell death. These emerging areas underscore the need for multi-targeted approaches that integrate targeted therapies, immunotherapy, and agents modulating the tumor microenvironment. For example, combination strategies targeting oxidative stress, angiogenesis, and immune escape mechanisms are gaining traction as potential solutions to overcome the limitations of existing treatments (García-Mulero et al., 2021; Zou and Yaguchi, 2023).

To enhance treatment efficacy and overcome resistance in melanoma, future research should prioritize several critical areas. Investigating novel resistance mechanisms, including the interplay between MAPK reactivation, PI3K/AKT pathway, and alternative survival pathways, is essential for understanding resistance and identifying predictive biomarkers for combination therapies. Advancing precision medicine through molecular profiling and leveraging multi-omics data will enable the development of personalized regimens. Artificial intelligence (AI) has been instrumental in precision medicine by identifying biomarkers like PD-L1 expression and tumor mutational burden, improving patient stratification for immune checkpoint inhibitors. AI models have also predicted resistance mechanisms to BRAF/MEK inhibitors by integrating multi-omics data and have optimized combination therapy regimens through patient-specific response simulations. Targeting the tumor microenvironment by modulating immunosuppressive components, such as Tregs and MDSCs, and exploring the roles of cytokines and exosomes, offers additional therapeutic opportunities. Multi-modal strategies, including the combination of immune checkpoint inhibitors with agents targeting ferroptosis, pyroptosis, or autophagy, as well as triple-combination regimens integrating targeted therapies and cell death modulators, hold promise for achieving more durable responses. As the field evolves, interdisciplinary collaboration and emerging technologies will be critical in advancing personalized and multi-faceted therapeutic strategies to address resistance in melanoma.

This study offers important insights into the research on BRAF and MEK inhibitor resistance in melanoma, but certain limitations should be noted. The reliance on the Web of Science Core Collection may exclude non-English publications, non-indexed journals, and emerging research with limited citations, potentially overlooking significant contributions. While this analysis spans 2 decades, the retrospective nature of the data requires ongoing updates to capture rapidly evolving research trends. Furthermore, the absence of molecular and clinical trial data limits the contextualization of findings within translational and therapeutic frameworks, potentially affecting the generalizability of conclusions. Future studies should broaden data sources and integrate experimental and clinical data to enhance the precision and relevance of bibliometric analyses, better addressing the complexities of resistance mechanisms and guiding innovative treatment strategies.

## Conclusion

This bibliometric analysis provides valuable insights into the global research landscape of BRAF and MEK inhibitor resistance in melanoma, offering a comprehensive understanding of key contributors, research trends, and emerging therapeutic strategies. This study not only maps the current state of the field but also serves as a vital resource for guiding future research, fostering innovation, and ultimately contributing to the development of more effective, personalized treatment strategies for melanoma patients.

## Data Availability

The original contributions presented in the study are included in the article/supplementary material, further inquiries can be directed to the corresponding author.
